# Improved production of recombinant β-mannanase (TaMan5) in *Pichia pastoris* and its synergistic degradation of lignocellulosic biomass

**DOI:** 10.3389/fbioe.2023.1244772

**Published:** 2023-09-07

**Authors:** Fengzhen Zheng, Abdul Basit, Zhiyue Zhang, Huan Zhuang, Jun Chen, Jianfen Zhang

**Affiliations:** ^1^ College of Biological and Environmental Engineering, Zhejiang Shuren University, Hangzhou, China; ^2^ Department of Microbiology, University of Jhang, Jhang, Pakistan; ^3^ Department of ENT and Head and Neck Surgery, The Children’s Hospital Zhejiang University School of Medicine, Hangzhou, Zhejiang, China; ^4^ Interdisciplinary Research Academy, Zhejiang Shuren University, Hangzhou, China

**Keywords:** *Trichoderma asperellum*, β-mannanase, improved production, transglycosylation, catalysis mechanism, synergism

## Abstract

Mannan, a highly abundant and cost-effective natural resource, holds great potential for the generation of high-value compounds such as bioactive polysaccharides and biofuels. In this study, we successfully enhanced the expression of constructed GH5 β-mannanase (TaMan5) from *Trichoderma asperellum* ND-1 by employing propeptide in *Pichia pastoris*. By replacing the α-factor with propeptide (MGNRALNSMKFFKSQALALLAATSAVA), TaMan5 activity was significantly increased from 67.5 to 91.7 U/mL. It retained higher activity in the presence of 20% ethanol and 15% NaCl. When incubated with a high concentration of mannotriose or mannotetraose, the transglycosylation action of TaMan5 can be detected, yielding the corresponding production of mannotetraose or mannooligosaccharides. Moreover, the unique mechanism whereby TaMan5 catalyzes the degradation of mannan into mannobiose involves the transglycosylation of mannose to mannotriose or mannotetraose as a substrate to produce a mannotetraose or mannopentose intermediate, respectively. Additionally, the production of soluble sugars from lignocellulose is a crucial step in bioethanol development, and it is noteworthy that TaMan5 could synergistically yield fermentable sugars from corn stover and bagasse. These findings offered valuable insights and strategies for enhancing β-mannanase expression and efficient conversion of lignocellulosic biomass, providing cost-effective and sustainable approaches for high-value biomolecule and biofuel production.

## 1 Introduction

Mannans, a prevalent sugar component found in hemicellulose, offer significant potential for the production of valuable derivatives such as prebiotic mannooligosaccharides (MOS) (Dhawan and Kaur, 2007; Do et al., 2009). These polysaccharides are connected by a β-1,4-linked mannose backbone decorated by diverse substituents (Sanchez, 2009). Several processes have been developed for the efficient degradation and conversion of mannans, including alkaline wet oxidation, enzymatic hydrolysis, and dilute acid hydrolysis (Moreira and Filho, 2008; Li et al., 2018; Jana et al., 2021). Enzymatic hydrolysis of mannans is a sustainable and environmentally friendly reaction, since it occurs under mild conditions and does not involve the production of harmful or toxic byproducts (Zhou et al., 2018; Liu Z et al., 2020).

Mannanase plays a key role in the degradation of mannan to yield MOS, which has a great potential application in industrial fields (Zhao et al., 2011; Srivastava and Kapoor, 2017). However, low activities and high cost of β-mannanases are the crucial technological bottleneck increasing the conversion rate of mannan (Katrolia et al., 2012). Nowadays, various genetic engineering technologies have been used to achieve the efficient expression of target proteins in plant, yeast, and bacterial expression hosts (Liu S et al., 2020). Notably, recent advancements in protein engineering have facilitated the expression of β-mannanases in *Pichia pastoris*, paving the way for improved production and utilization of β-mannanases.

*P. pastoris* is a widely used microorganism for the expression of heterologous proteins due to its well-established secretion pathway with little endogenous proteins (Do et al., 2009). Moreover, several approaches have been successfully performed to increase the expression of target proteins in *P. pastoris*, including codon optimization, gene copy number, and signal peptide sequences (Looser et al., 2015; Yang and Zhang, 2018; Kaira and Kapoor, 2021). Given its significant advantages, *P. pastoris* is a highly desirable platform for the high-level expression of β-mannanases.

In our previous investigation, we successfully expressed the GH5 β-mannanase (TaMan5) gene from *Trichoderma asperellum* ND-1 in *P. pastoris* and obtained a great specific activity of 718.14 U/mg (Zheng et al., 2023). However, the secretion of β-mannanase into the fermentation supernatant was insufficient. Hence, this study aimed to further enhance the TaMan5 yield by manipulating the N-terminal peptide in *P. pastoris*. We also investigated the transglycosylation of TaMan5 on mannotriose/mannotetraose and elucidated the novel catalytic mechanism underlying mannobiose production. Additionally, the synergistic effects of TaMan5 coupled with cellulase and xylanase were extensively explored on the degradation of natural lignocellulosic biomass, including corn stover and bagasse.

## 2 Materials and methods

### 2.1 Reagents

Mannooligosaccharides (M6, mannohexaose; M5, mannopentaose; M4, mannotetraose; M3, mannotriose; and M2, mannobiose) were obtained from Megazyme (Wicklow, Ireland). Locust bean gum (LBG) and β-xylanase (XYL) from *Thermomyces lanuginosus* and commercial cellulase (CEL) from *Trichoderma reesei* (ATCC 26921) were obtained from Sigma-Aldrich (USA).

### 2.2 Construction of recombinant plasmids and strains

The complete β-mannanase gene (*pman*) was discovered in the genome sequence of *T. asperellum* ND-1. By utilizing SignalP-5.0 software (http://www.cbs.dtu.dk/services/SignalP/), we predicted that TaMan5 possesses a 27-residue signal peptide (MGNRALNSMKFFKSQALALLAATSAVA). To optimize the full length for yeast codon preference (http://www.jcat.de/), *pman* gene was synthesized and then ligated to the pPICZαA vector (forming pPICZαp-*oman*) by Tsingke Corp. (Beijing).

Enzyme activity of TaMan5 was further increased by replacing α-factor with propeptide in *P. pastoris*. The recombinant plasmids, pPICZp-*oman* (with a propeptide sequence) and pPICZα-*oman* (with an α-factor signal peptide), were cloned from the synthesized vector pPICZαp-*oman* with the corresponding primer pairs (pPICZp-oman-F/pPICZp-oman-R and pPICZα-oman-F/pPICZα-oman-R) (Supplementary Table S1). The expression vectors, pPICZp-*oman* and pPICZα-*oman*, were linearized by *Sac*I, introduced into *P. pastoris* through electroporation, and screened based on the manufacturer’s protocol (Invitrogen, San Diego, CA). Transformants were grown on agar plates containing peptone dextrose (YPD) medium supplemented with 100 μg/mL Zeocin (Invitrogen, Carlsbad, CA, United States) to obtain one gene copy. The primer pair AOX-F/AOX-R was used to confirm the positive strains by PCR. The constructed strains were designated as α-oTaMan5 and p-oTaMan5.

### 2.3 Protein expression and SDS-PAGE

To produce β-mannanase TaMan5, recombinant strains of α-oTaMan5, p-oTaMan5, and X-33 were incubated in BMMY medium as previously reported (Zheng et al., 2018). All strains were grown at 30°C, with shaking at 200 rpm for a duration of six days. Throughout the fermentation period, the methanol concentration was maintained at 1.0 by supplementing with 100% (v/v) methanol (Basit et al., 2018). The fermentation broth was subjected to centrifugation (4°C, 5,000 rpm) to collect crude enzyme. The TaMan5 expression was analyzed in 12% SDS-PAGE, and its concentration was analyzed based on the Bradford method (Bradford, 1976). All media and operation steps were executed following the guidelines provided in the *P. pastoris* expression manual (Invitrogen, San Diego, CA).

### 2.4 Activity assay

TaMan5 activity was tested using 3,5-dinitrosalicylic acid (DNS) (Miller, 1959) at a temperature of 65°C, with LBG serving as the substrate. The reaction mixture consisted of 0.45 mL sodium acetate buffer (pH 4.0) containing 0.05 mL appropriate diluted enzyme and 0.5% (w/v) LBG. The reaction was terminated by adding 0.75 mL DNS and boiling the mixture for 5 min. The absorbance of the solution was assayed at 540 nm, with one unit (U) activity regarded as the amount of enzyme capable of liberating 1 μmol of reducing sugars per minute.

### 2.5 Effect of NaCl and ethanol on TaMan5 activity

The impact of various concentrations of NaCl and ethanol on the activity of TaMan5 was evaluated. TaMan5 was subjected to a 1-h incubation at 4°C in 50 mM sodium acetate solution (pH 4.0) with varying final concentrations of NaCl or ethanol, ranging from 0% to 20% (w/v). After incubation, residual enzymatic activity was separately assayed and compared with the control (without any additives). All assays were analyzed in triplicate.

### 2.6 Transglycosylation

Transglycosylation of TaMan5 toward mannotriose/mannotetraose was carried out by incubating 50 mg/mL mannotriose/mannotetraose with 1 U of TaMan5. At various time points, hydrolytic products (25 μL) were collected, boiled for 10 min, and then analyzed using thin-layer chromatography (TLC) following the method described by Katrolia et al. (2013). Substrates containing inactive enzymes were used as blank controls.

For TLC analysis, silica gel plates (Merck Silica Gel 60 F_254_, Germany) were used for separation, with a mobile phase consisting of n-propanol/ethanol/water (7:1:2, v/v/v). After development, the saccharides on the plates were visualized by immersing them in a solution of 5% sulfuric acid in methanol, followed by heating at 100°C for 15 min. Mannooligosaccharides (DP 2–6) and mannose were employed as the standard for the comparison and identification of the products.

### 2.7 Synergistic action

Lignocellulosic biomass (bagasse and corn stover) was pretreated by using an alkaline method as previously reported (Basit et al., 2018). The enzymatic degradation of 5% (w/v) corn stover or bagasse by CEL (15 U/g biomass), XYL (47 U/g biomass), and TaMan5 (55 U/g biomass) individually or in combination was performed at 50°C, with constant mixing for 72 h at 200 rpm. Blank controls containing only the substrate were also performed. After centrifugation for 5 min at 12,000 rpm, the supernatants were obtained, and the quantification of reducing sugars released from the biomass was assayed using the DNS method (Miller, 1959).

## 3 Results and discussion

### 3.1 Improved expression of TaMan5

Several strategies have been employed to achieve optimal expression levels of heterologous proteins in *P. pastoris* (Looser et al., 2015). Utilizing the secretion pathway of *P. pastoris*, which limits endogenous protein secretion, is considered an effective and desirable approach for recombinant protein production (Šiekštelė et al., 2015). The selection of appropriate signal peptide sequences has been recognized as a crucial factor in enhancing protein expression in *P. pastoris*, focusing on improving the secretion level and recruitment of different signal peptides (Vadhana et al., 2013; Zheng et al., 2018; Püllmann and Weissenborn, 2021).

**FIGURE 1:**
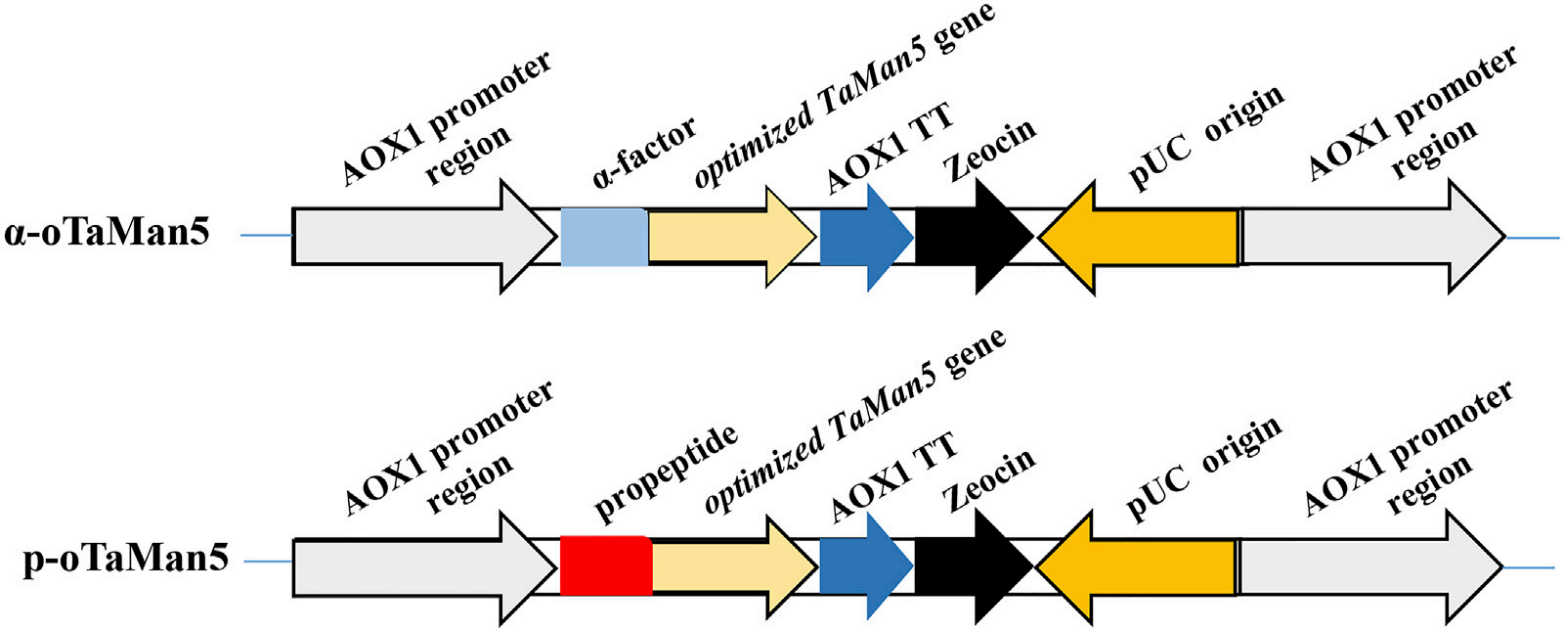
Schematic representation of recombinant strains α-oTaMan5 and p-oTaMan5.

To enhance the enzyme activity of TaMan5, the α-factor was substituted with a propeptide. Two different recombinant vectors, pPICZp-*oman* (containing the propeptide sequence) and pPICZα-*oman* (containing the α-factor signal peptide), were designed. The recombinant vectors were transformed into *P. pastoris*, and engineering strains, α-oTaMan5 and p-oTaMan5 (Figure 1), were selected on YPD medium supplemented with Zeocin (100 μg/mL).

**FIGURE 2:**
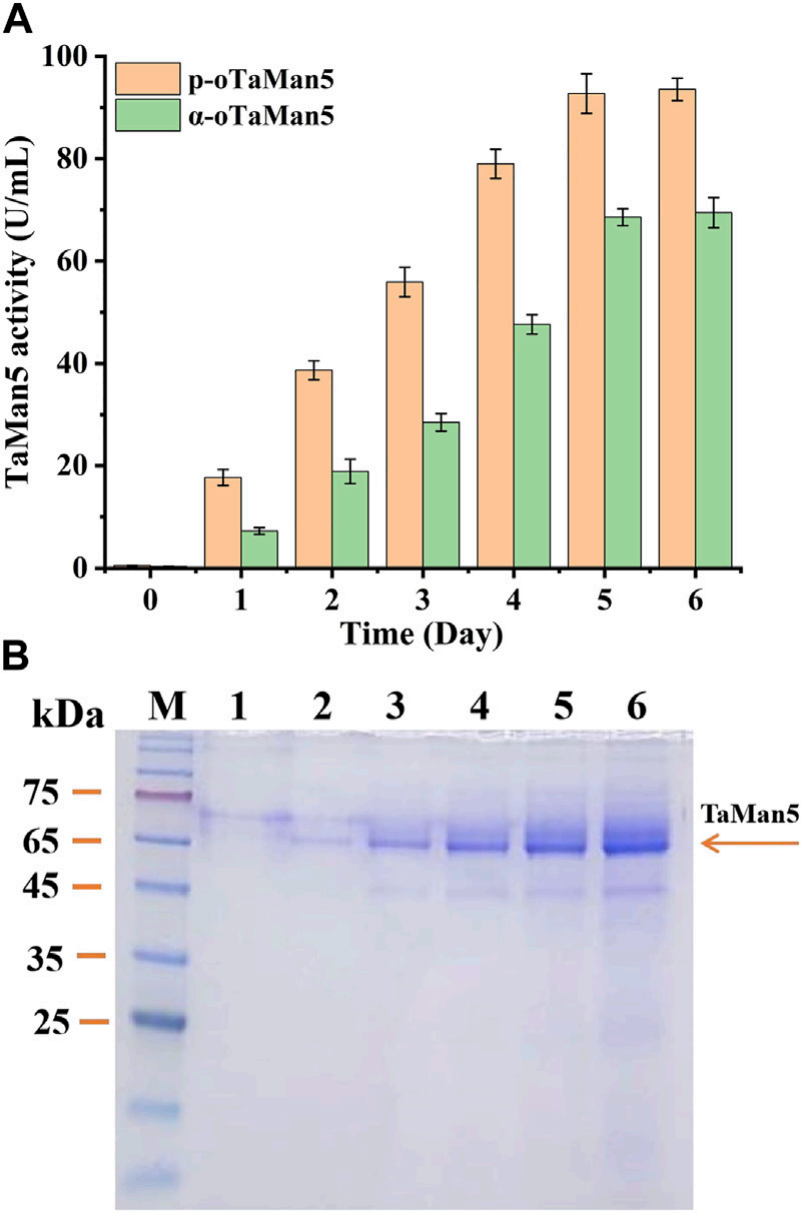
High-efficiency expression of TaMan5 in *Pichia pastoris*. **(A)** Extracellular enzyme activity of recombinant strains α-oTaMan5 and p-oTaMan5. **(B)** SDS-PAGE analysis of proteins expressed by p-oTaMan5. 1, 24 h; 2, 48 h; 3, 72 h; 4, 96 h; 5, 120 h; and 6, 144 h.

Following induction with 1% (v/v) methanol for 6 days, TaMan5 activity of the recombinant strain p-oTaMan5 (with the propeptide sequence) continuously increased, reaching a maximum of 93.56 ± 2.17 U/mL (779.67 ± 18.08 U/mg) (Figure 2A), which was remarkably 1.35-fold higher than that of α-oTaMan5 (with the α-factor signal peptide). Previous studies have demonstrated that the recruitment of natural propeptides, such as trypsin from *Streptomyces griseus* (Zhang et al., 2018) and α-L-arabinofuranosidase from *Aspergillus niger* ND-1 (Zheng et al., 2018), can remarkably enhance the production levels of heterologous proteins in *P. pastoris*. SDS-PAGE results revealed that the recombinant proteins secreted by p-oTaMan5 have the molecular weight of approximately 66 kDa (Figure 2B). Protein expression in yeast is transferred through a secretory system from the endoplasmic reticulum to Golgi bodies and finally to the extracellular space (Nett et al., 2011). Taken together, these findings suggested that the recruitment of a propeptide is an effective and potential strategy for improving TaMan5 activity.

### 3.2 Effect of NaCl and ethanol on the activity of TaMan5

**FIGURE 3:**
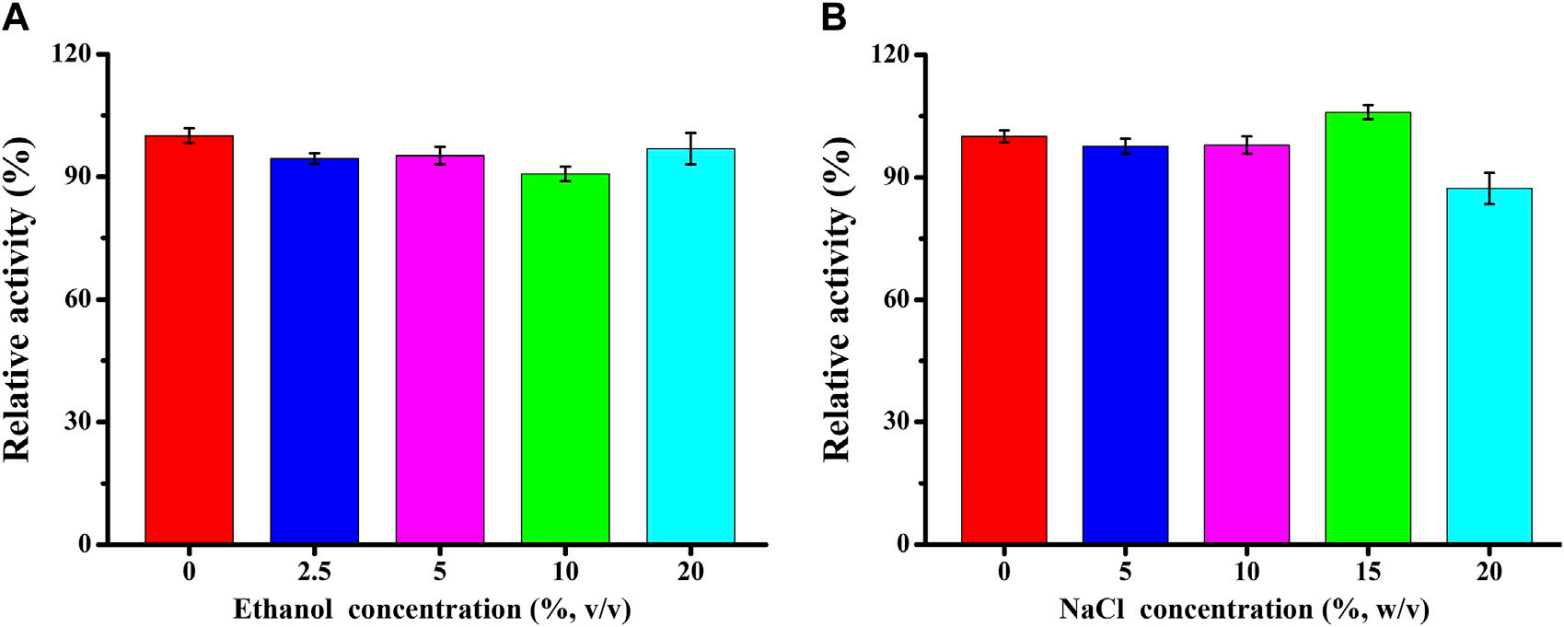
Effect of varying ethanol **(A)** and NaCl **(B)** concentrations on TaMan5 activity.

The impact of varying concentrations of ethanol and NaCl on TaMan5 activity was evaluated (Figure 3). TaMan5 retained nearly full activity across a wide range of ethanol concentrations (0%–20%) (Figure 3A), whereas rManAJB13 from *Sphingomonas* sp. JB13 retained 69.1% of the β-mannanase activity at 10% of ethanol concentration (Zhou et al., 2012). Ethanol-tolerant β-mannanases could potentially be applied in bioethanol production, and TaMan5 has potential application in the process of biocatalysts. Similar to other hemicellulases, including β-mannanases (Jiang et al., 2006) and β-xylanases (Yegin, 2017), TaMan5 exhibited higher activity at 15% of salt concentration (Figure 3B), suggesting that it might be a very beneficial additive in the processing of seafoods and saline foods, such as sauces and pickles.

### 3.3 Catalytic mechanism of TaMan5

**FIGURE 4:**
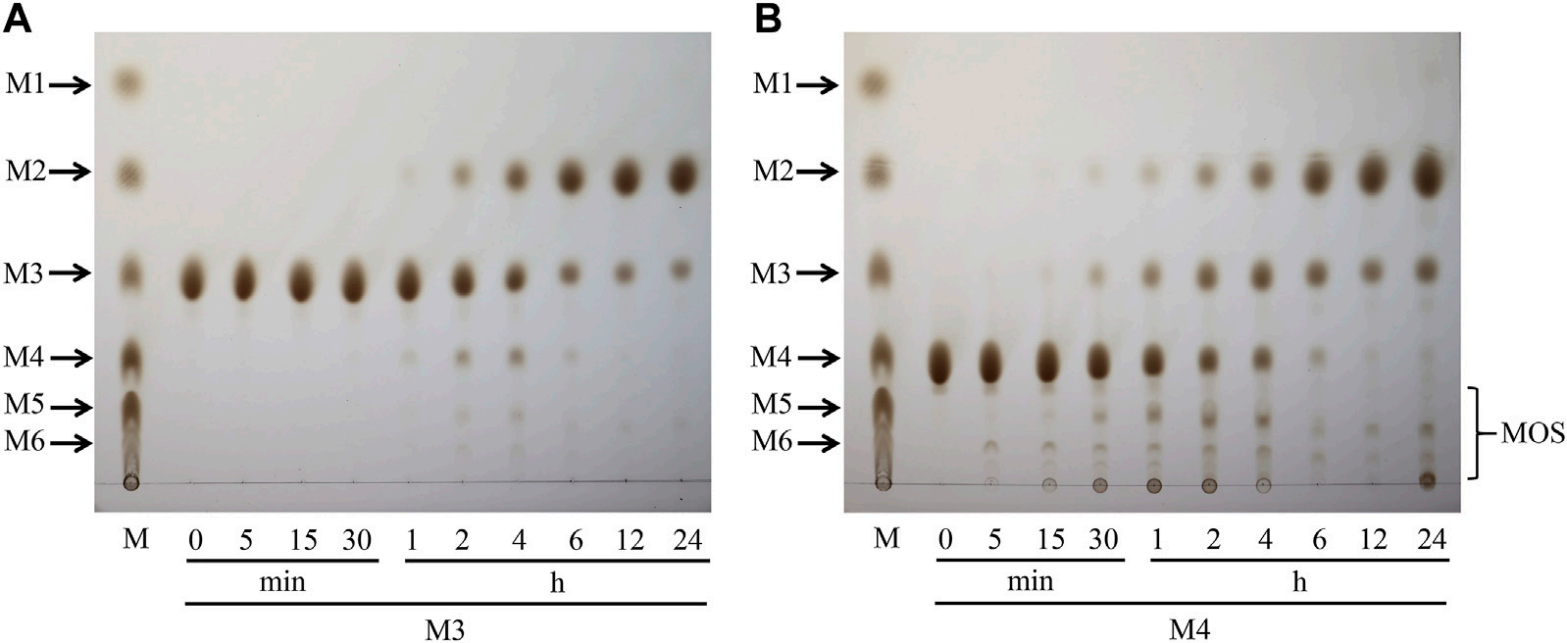
Transglycosylation reaction of TaMan5 by TLC analysis. **(A, B)** Time-course hydrolysis of mannotetraose and mannotriose by TaMan5. Mannohexaose (M6), mannopentaose (M5), mannotetraose (M4), mannotriose (M3), mannobiose (M2), and mannose (M1). Substrates containing inactive enzymes were used as blank controls (0 min).

**FIGURE 5:**
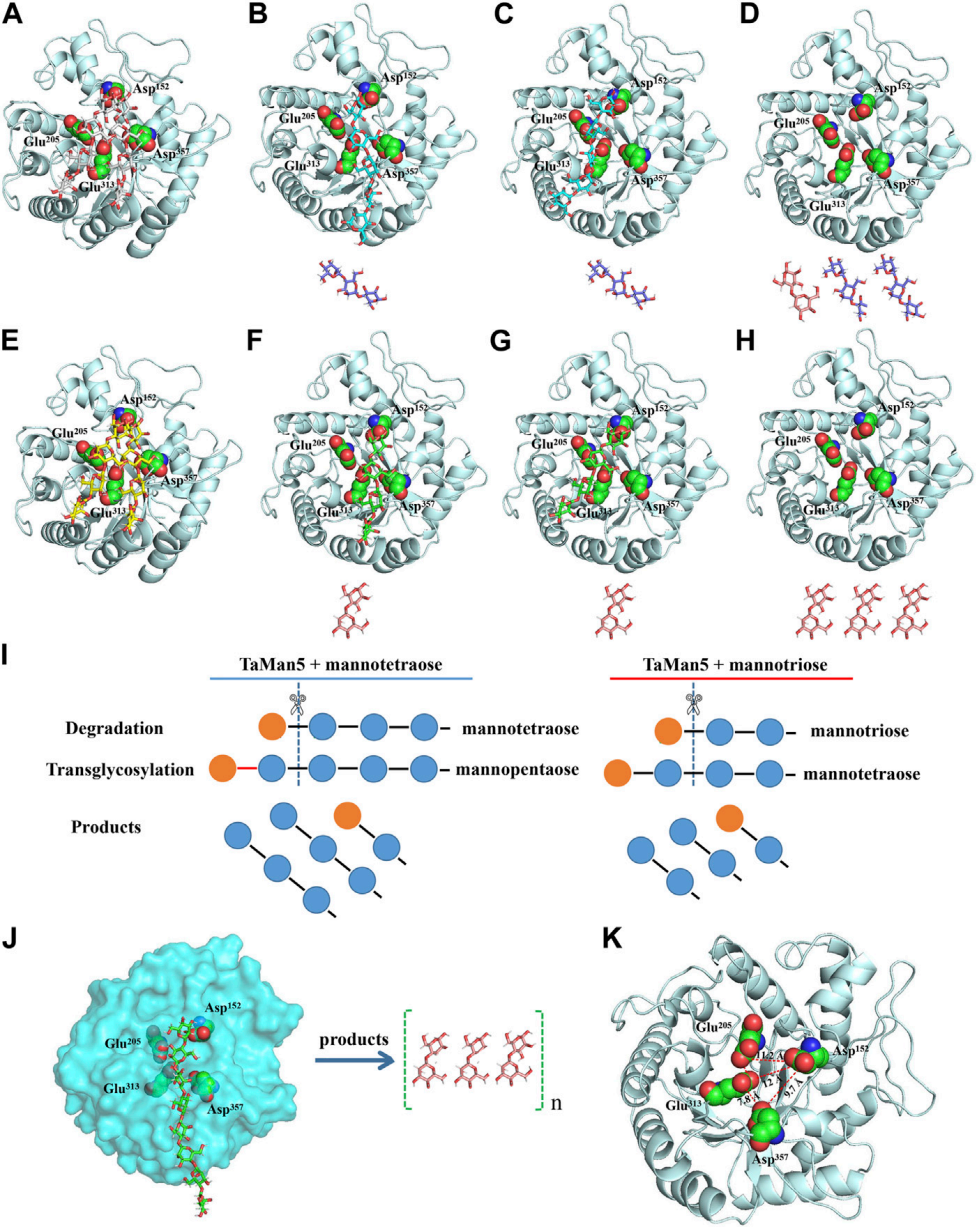
Catalytic mechanism of TaMan5. **(A–H)** Molecular docking studies of TaMan5 showing substrates and intermediate products in predicted active sites during the hydrolysis of mannotetraose **(A–D)**, mannotriose **(E–H)**, and mannan **(J)**. **(I)** Schematic model of the hydrolysis mechanism of mannotriose and mannotetraose by TaMan5. **(K)** Enzyme structure of the catalytic region showing distances (Å) among catalytic residues.

Hydrolysis properties of TaMan5 showed that it required at least three mannose (mannotriose) residues as the substrate for the production of mannobiose as described previously (Zheng et al., 2023). Mannotriose was completely hydrolyzed into mannobiose, and no mannose was detected, indicating that TaMan5 exhibited transglycosylation as well as hydrolysis function. Moreover, when incubated with a high concentration of mannotriose (Figure 4A) or mannotetraose (Figure 4B), the transglycosylation action of TaMan5 can be detected, yielding the corresponding production of mannotetraose or MOS (DP > 4) (Figure 4). Interestingly, Asp^152^ is positioned opposite to the enzyme’s active region (Glu^313^ and Glu^205^) based on the 3D structure of TaMan5 (Figure 5A). Thus, we deduced that Asp^152^ is probably responsible for the binding of substrates to catalytic residues. Moreover, the distance between crucial catalytic sites was determined using PyMOL software, and the inter-residue distances of Glu^205^–Glu^313^ and Glu^313^–Asp^357^ are ∼4.5 and 7.8 Å, respectively (Figure 5K), which was much shorter than those of Glu^205^–Asp^152^ (11.2 Å) and Glu^313^-Asp^152^ (12.0 Å) (Figure 5K). Based on 3D structure analyses, mutant activity, and space distance, Asp^152^ can promote the combination of mannotriose/mannotetraose and active center, the catalytic sites (Glu^313^–Asp^357^) are responsible for transglycosylation action, and Glu^205^–Glu^313^ are crucial sites for hydrolysis function.

To better elucidate the action mode of TaMan5, molecular docking analysis was carried out (Figure 5). Mannotriose, mannotetraose, and mannan were docked into the catalytic region comprising Glu^205^, Glu^313^, Asp^152^, and Asp^357^ residues (Figures 5A–J). For mannotetraose, catalytic residues Glu^313^ and Glu^205^ of TaMan5 degrade mannotetraose into mannose and mannotriose (Figure 5B), and Glu^357^ and Glu^313^ then promote the formation of an intermediate (mannopentose) (Figure 5B) from new substrates (mannose and mannotetraose) for further hydrolysis by catalytic sites (Glu^313^ and Glu^205^) (Figure 5C), generating mannobiose and mannotriose (Figure 5C). For mannotriose, Glu^313^ and Glu^205^ transform mannotriose into mannose and mannobiose (Figure 5F), and Glu^357^ and Glu^313^ then promote the formation of intermediate (mannotetraose) (Figure 5F) from new substrates (mannose and mannotriose) for further degradation (Figure 5G), generating mannobiose (Figure 5H). The catalytic mechanism of TaMan5 involves the transglycosylation of mannose to mannotriose or mannotetraose as the substrate to produce a mannotetraose or mannopentose intermediate, respectively (Figure 5I). Based on hydrolysis and transglycosylation process of other β-mannanases reported previously (Jiang et al., 2006; Do et al., 2009; Katrolia et al., 2013), this study proposed a novel degradation process of mannotetraose: 2 M4 + E = 2 M4(E); 2 M4(E) = M3 + (M1 + M4)(E); (M1 + M4)(E) + M3 = M5(E) + M3; M5(E) + M3 = M2 + 2 M3 + E, where 2 M4 represents two mannotetraoses; E, free TaMan5; M4(E), TaMan5–mannotetraose complex; M3, mannotriose; (M1 + M4) (E), mannose–mannotetraose–TaMan5 complex; M1, mannose; M5(E), TaMan5–mannopentose complex; M5, mannopentose; M2, mannobiose; M3, mannotriose; and 2 M3, two mannotrioses. For mannotriose, 2 M3 + E = 2 M3(E); 2 M3(E) = (M1 + M3)(E) + M2; (M1 + M3)(E) + M2 = M4(E) + M2; M4(E) + M2 = 3 M2 + E (where M3(E) represents the TaMan5–mannotriose complex; (M1 + M3)(E), mannose–mannotriose–TaMan5 complex; 3 M2, three mannobioses; and other abbreviations as mentioned previously), which are consistent with TLC results, 3D structure, site-directed mutagenesis, and space distance of crucial catalytic sites Glu^205^, Glu^313^, and Asp^357^ in the degradation of mannan to produce mannobiose. These results illustrate the diverse functionality of GH5 β-mannanases from fungi and offer valuable reference data to guide future advancements in enzyme engineering and biocatalysis.

### 3.4 Synergistic action

**FIGURE 6:**
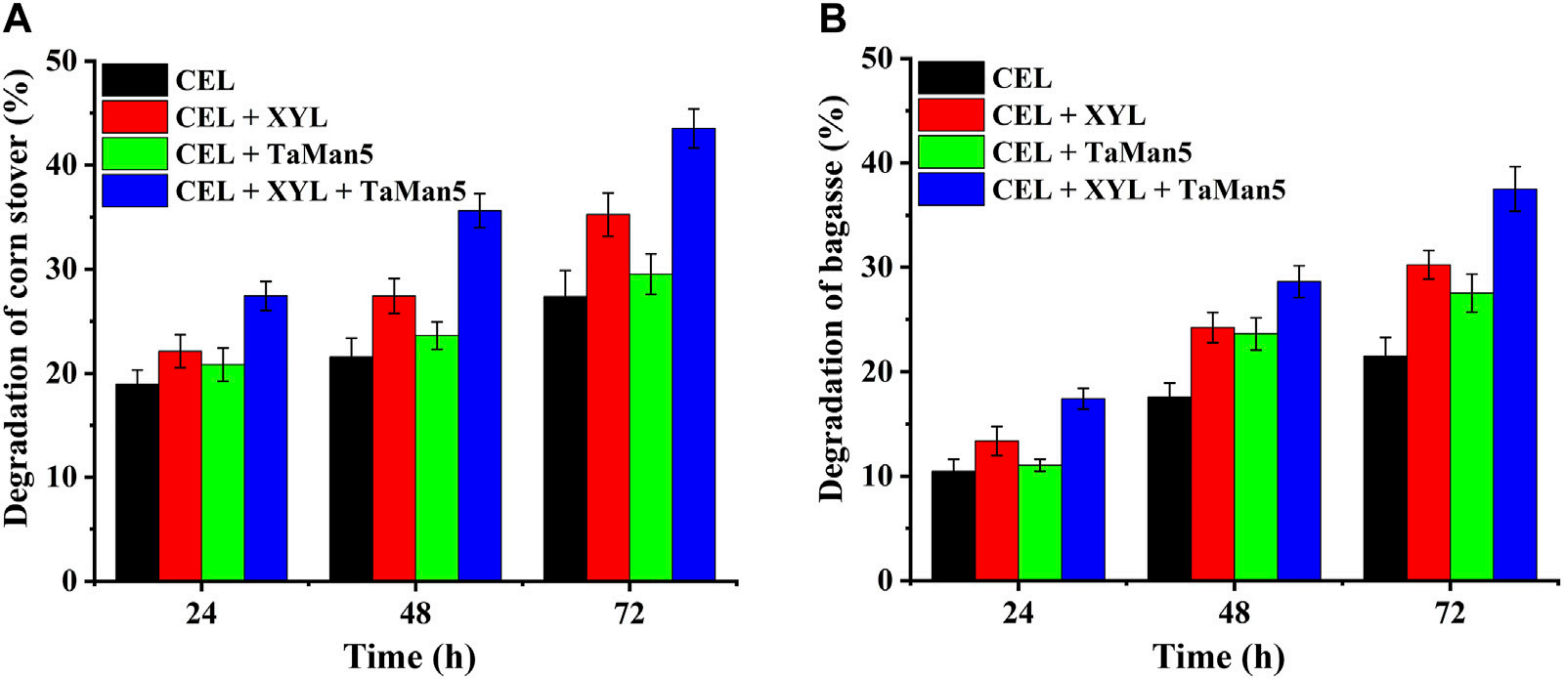
Synergistic action of TaMan5 with the addition of commercial cellulase (CEL) and β-xylanase (XYL) on the degradation of corn stover **(A)** and bagasse **(B)**. Data are presented as the mean ± SD (*n* = 3).

In natural systems, the efficient breakdown of biomass polysaccharides relies on the cooperative action of diverse carbohydrate-active enzymes (CAZymes), particularly cellulases and hemicellulases (Gao et al., 2011; Bhattacharya et al., 2015; Mattam et al., 2022). As shown in Figure 6, the addition of CEL (15 U/g biomass) resulted in the degradation of approximately 21.5% and 27.4% of bagasse and corn stover, respectively, following a 72-h incubation at 50°C and pH 4.0. When xylanase and mannanase were supplemented alongside cellulases, a synergistic effect was observed, leading to significantly improved saccharification levels. Specifically, the saccharification levels increased to 43.5% for corn stover (Figure 6A) and 37.5% for bagasse (Figure 6B). This enhanced saccharification efficiency highlights the cooperative action of these enzymes and underscores their importance in biomass degradation processes.

Furthermore, the enzymatic degradation level, also referred to as the conversion of reducing sugars, was significantly enhanced when XYL and TaMan5 were added together and was 58.8% and 42.7% higher than that of CEL alone toward corn stover and bagasse (Figure 6). The removal of hemicellulose and the improved degradation efficiency can exert a cooperative action by enhancing the accessibility of cellulases to cellulose. This, in turn, allows for the reduction of enzyme dosages, representing a sustainable approach for the production of value-added products from agricultural waste (Jeon et al., 2011; Moraïs et al., 2011; Várnai et al., 2011; Rakotoarivonina et al., 2016).

## 4 Conclusion

In this study, the expression of recombinant GH5 β-mannanase (TaMan5) from *T. asperellum* ND-1 was successfully enhanced by employing a propeptide in *P. pastoris*. It retained higher activity in the presence of 20% ethanol and 15% NaCl. When incubated with a high concentration of mannotriose or mannotetraose, the transglycosylation action of TaMan5 can be detected, yielding the corresponding production of mannotetraose or mannooligosaccharides. A proposed model illustrated the hydrolysis mechanism of mannan into mannobiose by TaMan5. Additionally, the combined action of TaMan5 with commercial cellulase and xylanase showed significant enhancement in the yield of fermentable sugars. All these excellent properties make TaMan5 a suitable candidate for various industrial applications, particularly in the valuable biomolecule production from agricultural residues.

## Data Availability

The original contributions presented in the study are included in the article/Supplementary Material. Further inquiries can be directed to the corresponding author.
