# Supplementary material for: Improved production of recombinant β-mannanase (TaMan5) in Pichia pastoris and its synergistic degradation of lignocellulosic biomass
Source: Front Bioeng Biotechnol. 2023 Sep 7;11:1244772. doi: 10.3389/fbioe.2023.1244772 (PMC10513448; doi:10.3389/fbioe.2023.1244772)
Supplement: Supplementary file 1 [file Table1.doc]

Supporting Information for:

**Improved production of recombinant β-mannanase (TaMan5) in *Pichia pastoris* and its synergistic degradation of lignocellulosic biomass**

**Authors:** Fengzhen Zheng^1^,*, Abdul Basit^2^, Zhiyue Zhang^1^, Huan Zhuang^3^, Jun Chen^4^, Jianfen Zhang^1^

**Affiliations:**

^1^ *College of Biological and Environmental Engineering, Zhejiang Shuren University,* *Hangzhou 310021, China.*

^2^ *Department of Microbiology, University of Jhang, Jhang 35200, Pakistan.*

^3^ *Department of ENT and Head & Neck Surgery, The Children's Hospital Zhejiang University School of Medicine, Zhejiang, Hangzhou, 310051, China.*

^4^ *Interdisciplinary Research Academy, Zhejiang Shuren University, Hangzhou 310021, China.*

***Corresponding author:** Fengzhen Zheng

**E-mail address:** [18811068358@163.com](mailto:18811068358@163.com)

**Present address:** College of Biological and Environmental Engineering, Zhejiang Shuren University, Hangzhou 310021, China.

**Table S1.** Oligonucleotide primers used in this study.

| **Primers** | **Sequence ( 5´- 3´ )** |
| --- | --- |
| AOX-F | GACTGGTTCCAATTGACAAGC |
| AOX-R | GCAAATGGCATTCTGACATCC |
| **TaMan5 variants** | |
| pPICZp-*oman*-F | AACTAATTATTCGAAACGATGGGAAACAGAGCCTTG |
| pPICZp-*oman*-R | CAAGGCTCTGTTTCCCATCGTTTCGAATAATTAGTT |
| pPICZα-*oman*-F | AAAAGAGAGGCTGAAGCTACTCCATTGCAATCCGTT |
| pPICZα-*oman*-R | AACGGATTGC AATGGAGTAG CTTCAGCCTC TCTTTT |
